# Dynamic thiol/disulfide homeostasis and oxidant status in patients with hypoparathyroidism

**DOI:** 10.2478/jomb-2019-0036

**Published:** 2020-01-23

**Authors:** Koca Arzu Or, Murat Dağdeviren, Tolga Akkan, İhsan Ateş, Salim Neşelioğlu, Özcan Erel, Mustafa Altay

**Affiliations:** 1 University of Health Sciences, Keçiören Health Administration and Research Centre, Department of Endocrinology and Metabolism, Ankara, Turkey; 2 University of Health Sciences, Numune Health Administration and Research Centre, Department of Internal Medicine, Ankara, Turkey; 3 University of Ankara Yıldırım Beyazıt, Department of Medical Biochemistry, Ankara, Turkey

**Keywords:** hypoparathyroidism, total oxidant status, total antioxidant status, disulfide, thiol, paraoxonase, paraoksanaza, tiol, disulfid, ukupni oksidacioni status, ukupni antioksidacioni status, hipoparatiroidizam

## Abstract

**Background:**

In this study, we aimed at determining the dynamic thiol/disulfide homeostasis and oxidant balance, and investigating the relation of these parameters to the severity of the disease and the serum calcium levels.

**Methods:**

55 patients with iatrogenic hypoparathyroidism follow-ups and 40 healthy volunteers were included in the study. The blood dynamic thiol/sulfide balance, Total Antioxidant Status (TAS), Total Oxidant Status (TOS), Paraoxonase Enzyme Activity (PON) levels were measured in serum samples.

**Results:**

In our study, it was found that the disulfide, disulfide/native thiol, disulfide/total thiol levels were higher in the hypoparathyroidism group. A negative correlation was found between 25-hydroxy vitamin D (25-OH vitamin D) and disulfide, disulfide/native thiol and disulfide/total thiol, and a positive correlation was found between native thiol and total thiol ratio; and the corrected calcium levels and PON levels were negatively correlated.

**Conclusions:**

Consequently, a change in favour of disulfide was found in the dynamic thiol-disulfide homeostasis in the hypoparathyroidism group in our study.

## Abstract (sr)

**Uvod:**

Cilj ovog istraživanja je bio da utvrdimo dinamičnu tiol/disulfidnu homeostazu i ravnotežu oksidanata, i istražimo odnos ovih parametara prema težini bolesti i nivoima kalcijuma u serumu.

**Metode:**

U ovu studiju bilo je uključeno 55 pacijenata sa jatrogenim hipoparatiroidizmom i 40 zdravih dobrovoljaca. U uzorcima seruma određivana je dinamička ravnoteža tiola/sulfida u krvi, ukupni antioksidacioni status (TAS), ukupni oksidacioni status (TOS), aktivnost enzima paraoksonaze (PON).

**Rezultati:**

U našoj studiji je utvrđeno da su nivoi disulfida, disulfida/prirodnog tiola, disulfida/ukupnog tiola bili viši u grupi disulfida/nativnog tiola i disulfida/ukupnog tiola, i utvrđena je pozitivna korelacija između odnosa prirodnog tiola i ukupnog tiola; a korigovani nivoi kalcijuma i nivoi PON bili su negativno povezani.

**Zaključak:**

Shodno tome, došlo je do promene u korist disulfida u dinamičkoj tiol disulfidnoj homeostazi u grupi hipoparatiroidizma u našoj studiji.

## Introduction

Hypoparathyroidism is an endocrine disorder caused by insufficient parathormone (PTH) secretion or, very rarely, by PTH receptor resistance. The most frequent cause for parathyroidism in adults is the neck operations. It generally progresses with hypocalcemia, hyperphosphatemia, and low-normal PTH levels [1]. While the electrolyte disorder developing in hypoparathyroidism has short-term symptomatic effects, there is also an increase in atherosclerosis and ischemic heart disease risks due to the pathological changes in the endothelium and cardiovascular system in the long run [2].

Hyperphosphatemia induced endothelium damage can generally be caused by oxidant radicals increasing in several mechanisms due to the decrease in the release of nitric oxide [3]. In addition to this, an increase in both chronic inflammation and reactive oxygen radicals can be observed with the deteriorating systemic perfusion due to long-term hypocalcemia induced cardiac depression [4].

Temporarily increased free oxygen radical concentrations play a crucial role in maintaining the homeostasis of the organism. Because of that, increased oxygen radicals comprise a part of the system in inflammatory events. However, free radicals are rather labile and reactive molecules. Long-lasting excessive free oxygen radical levels may cause DNA damage, oxidation of lipids and proteins, and cell damage [5] [6] [7]. Materials that prevent or recede the oxidation of these biological materials present in the living cells are called the antioxidants. The oxidative balance shows the equilibrium between the production rate of free radicals and the collapse rate. With this balance, the damage to the organism by the free radicals is prevented. The imbalance between free radical production and the antioxidant defence system for free radicals is called the »oxidative stress« [7] [8].

Total antioxidant status (TAS) reflects the total effect of all antioxidants, and total oxidant status (TOS) reflects the total effect of all oxidants in plasma and body fluids [7] [9] [10]. Paraoxonase 1 (PON1) is a serum esterase and the most known member of the paraoxonase multigene family [11]. The main function of PON1 is to protect the low-density lipoprotein cholesterol (LDL-c) and the high-density lipoprotein cholesterol (HDL-c) from oxidation and thus prevent cardiovascular diseases and atherosclerosis [12].

Oxidant radicals form disulfide (-S-S-) bonds by causing the oxidation of thiol groups of proteins comprised of amino acids that contain sulphur. These disulfide bonds are the earliest indicators of protein oxidation, and they are reversible. Decreasing the reversible disulfide bonds in thiol groups plays a role in maintaining dynamic thiol/disulfide (DT/DS) homeostasis. Dynamic thiol/disulfide balance has a critical part in antioxidant protection, detoxification, apoptosis, and the regulation of enzymatic activity. Therefore, it is significant that this balance is protected [13].

The sufficient data on whether hypoparathyroidism causes oxidative stress and what could its clinical reflections be are not present in the literature yet. However, it is thought that hypocalcemia and/or low PTH levels could cause several clinical issues by inducing oxidative stress. Therefore, we aimed at evaluating the new-generation oxidant status indicators and the oxidant-antioxidant levels in patients with hypoparathyroidism.

## Materials and Methods

Seventy patients between 18 and 65 years of age (n=70), who came to the Sa lık Bilimleri University Keçiören Training and Research Hospital Endocrinology and Metabolism Diseases polyclinic in 2017 and 2018, who were diagnosed with hypoparathyroidism, and 70 healthy controls (n=70) without parathyroidism were included in the research.

Patients with hyperparathyroidism, with any hematological or solid organ malignity, with active rheumatism, who had myocardium infarct or cerebrovascular event in the last year, who had chronic kidney disease, who had organ transplants, who were immunosuppressive, who had 25 OH vitamin D levels below 24.9 nmol/L, and who smoked and/or used alcohol were excluded from the study.

The age, sex, height, weight, drugs used, additional diseases, hypocalcemia symptoms, and hypoparathyroidism causes were recorded for all patients, their detailed anamneses were taken and the routine physical examinations were carried out. The PTH (18-88 ng/L), calcium (Ca) (2.1-2.5 mmol/L), phosphorus (P) (0.80-1.45 mmol/L), magnesium (Mg) (0.20-1.23 mmol/L), albumin (Alb) (35-52 g/L), alkaline phosphatase (ALP) (30-120 U/L), Blood Urea Nitrogen (BUN) (2.74-7.03 mmol/L), creatinine (58.3-97.2 μmol/L), 25 OH Vitamin D (74.8-249.6 nmol/L), and C reactive protein (CRP) (4.76-47.6 nmol/L) levels were recorded.

Ten hours after the physical examination of the participants of the study, the blood samples were taken, they were centrifuged, and the serum parts were separated and stored at 80°C. The blood dynamic thiol/disulfide balance, Total Antioxidant Status (TAS), Total Oxidant Status (TOS), and Paraoxonase Enzyme Activity (PON), Oxidative Stress Index (OSI) levels were examined in the serum samples.

The oxidative stress index was calculated using the formula TOS (μmol H_2_O_2_ equivalent/L)/TAS (mmol Trolox equivalent/L) [14], the corrected calcium using the formula »(4-serum albumin) x 0.8 + serum calcium«, and the body mass index (BMI) using the formula (kg)/boy^2^ (m^2^).

Serum TOS level was measured with a commercial kit (Rel Assay Diagnostics, Turkey, REF. No: RL0024, LOT No: JE 14048Og) via colorimetric method; CV%: 10. Linearity: 0-33.5 μmol/L. The results are expressed in micromolar H_2_O_2_ equivalents per litre (mmol/L H_2_O_2_ equivalent/L) [10].

Serum TAS level was measured with a commercial kit (Rel Assay Diagnostics, Turkey, REF. No: RL0017, LOT No: JE 14042 A) via colorimetric method; CV%: 10. Linearity: 0-2.75 mmol/L. The results are expressed in mmol Trolox equivalent/L (mmol Trolox equivalent/L) [9].

Serum PON1 level was measured with a commercial kit (Rel Assay Diagnostics, Turkey, REF. No: RL0031, LOT No: JE14028P) via colorimetric method; CV%: 5. Linearity: 0-750 U/L. PON1 activity was expressed as U/L of serum [11].

Thiol/Disulfide homeostasis tests were performed as previously described [10]. Briefly, reducible disulfide bonds were first reduced to form free functional thiol groups. Unused reductant sodium borohydride was consumed and removed with formaldehyde, and all of the thiol groups, including reduced and native thiol groups were determined after the reaction with DTNB (5,5’-dithiobis-(2-nitrobenzoic acid). Half of the difference between the total thiols and native thiols gave the dynamic disulfide amount. After the determination of native and total thiols, disulfide amounts, disulfide/total thiol percent ratios, native thiol/total thiol percent ratios, and disulfide/native thiol percent ratios were calculated [15].

### Compliance with ethical standards

The ethical committee approval was obtained from the Sa lık Bilimleri University, Keçiören Training and Research Hospital, Clinical Ethics Committee (Project No: KAEK-15/1696).

### Ethical approval

All procedures performed in studies involving human participants were in accordance with the ethical standards of the institutional and/or national research committee and with the 1964 Helsinki Declaration and its later amendments or comparable ethical standards.

### Informed consent

Oral and written informed consents were taken from all patients included in the study.

### Statistical Analysis

The data obtained in this study were analysed using the IBM SPSS Statistics 22 software. Whether the data distributed normally or not was decided by using Kolmogorov-Smirnov/Shapiro-Wilk tests and the distribution of the data on the histogram charts. The normally distributed data were expressed as mean ± standard deviation, and the non-normally distributed data were expressed as median (minimum-maximum). Categorical variables were presented as percentages, and the statistical analyses were carried out using Chi-square test. To evaluate the differences between the groups, the Student t-test, and Mann-Whitney U test were used. When examining the correlations between the variables, the Spearman and Pearson correlation tests were used. The data were analysed with the multivariate linear regression model to determine the independent predictors to predict the oxidative stress parameters. The model fit was examined using the required residual and goodnessof-fit statistics. A statistically significant difference/ correlation was acknowledged in the case of p<0.05.

## Results

Severe vitamin D deficiency (<24.9 nmol/L) was found in 10 of the 70 patients included in the study in the hypoparathyroidism group, hypertension was found in three patients and diabetes in two patients, and the study was completed with 55 patients. In the healthy control group, on the other hand, severe vitamin D deficiency was found in 15 patients (<24.9 nmol/L), hypertension was found in nine patients and diabetes in six patients, and the study was completed with 40 healthy volunteers. Both groups were similar regarding age, sex, and BMI. The hypoparathyroidism was secondary (iatrogenic) to a neck operation in all individuals in the patient group. The calcium, corrected calcium (cCa) and PTH levels were found significantly lower, and the phosphor level was found significantly higher when compared to the control group (p<0.001) (Table 1).

**Table 1:** Demographic, characteristics, and the distribution of laboratory findings to the control and hypoparathyroid groups.(Categorical variables were shown in numbers and percentage; numerical variables were shown as mean ± S.D.) eGFR: Estimated Glomerular Filtration Rate, BMI: Body mass index, BUN: Blood Urea Nitrogen, ALP: Alkaline phosphatase, PTH: Parathormone, Mg: Magnesium, Ca: Calcium, cCa: Corrected calcium

	Control (n=40)	Hypoparathyroidism (n=55)	p
Gender, n (%)			NS
Female	34 (85)	49 (89.1)	
Male	6 (15)	6 (10.9)	
Age (years)	41.9±11.33	44.58±11.1	NS
BMI (kg/m^2^)	29.55±6.34	29.52±5.44	NS
BUN (mmol/L)	4.07±0.92	4.90±1.28	0.001
Creatinine (mmol/L)	66.3±9.72	68.06±8.84	NS
eGFR (mL/min/1.73 m^2^)	101.66±12.68	96.51±14.8	NS
ALP (U/L)	62 (25–112)	59 (30–224)	NS
Albumin (g/L)	44 (39–55.8)	44 (39–52)	NS
Ca (mmol/L)	2.38±0.08	2.08±0.19	<0.001
cCa (mmol/L)	2.28±0.10	1.97±0.19	<0.001
P (mmol/L)	1.07±0.16	1.54±0.34	<0.001
Mg (mmol/L)	0.88 (0.65–1.56)	0.78 (0.61–3.16)	NS
PTH (ng/L)	38.1 (17–63)	3 (3–32.8)	<0.001
25-OH D vitamin (nmol/L)	51.91 (26.38–162.98)	53.91 (11.23–208.16)	NS

In the analyses, the disulfide, disulfide/native thiol, disulfide/total thiol levels were found higher in the hypoparathyroidism group than the control groups (p=0.002, p=0.001, p=0.001, respectively); and the native thiol/total thiol level was found lower than the control group (p=0.001) (Table 2). In both groups, the TAS, TOS, OSI, PON, Native and Total thiol levels were found similar (Table 2).

**Table 2:** The level of all oxidative stress parameters according to groups OSI: Oxidative stress index, TAS: Total Antioxidant Status, TOS: Total Oxidant Status, PON: Paraoxonase Enzyme activity

	Control (n=40)	Hypoparathyroidism (n=55)	p
TAS (mmol Trolox equivalent/L)	1.78±0.3	1.83±0.18	NS
TOS (μmol H_2_O_2_ equivalent/L)	10.25±4.22	9.4±2.87	NS
OSI (arbitrary unit)	5.64±1.8	5.14±1.49	NS
PON (U/L)	357.5 (76.5–1033.1)	439.2 (118.4–1190.8)	NS
Native thiol (μmol/L)	425.09±64.45	435.38±51.22	NS
Total thiol (μmol/L)	460.43±60.52	475.52±54.97	NS
Disulfide (μmol/L)	17.25 (8.25–43.35)	19.7 (9.1–30.2)	0.002
Disulfide/Native thiol (%)	3.87 (1.71–20.16)	4.67 (2.66–7.23)	0.001
Disulfide/Total thiol (%)	3.6 (1.66–14.37)	4.27 (2.53–6.31)	0.001
Native thiol/Total thiol (%)	92.8 (71.26–96.69)	91.46 (87.37–94.94)	0.001

When all participants were examined, positive correlations between age and disulfide/native thiol and disulfide/total thiol ratios (p=0.007 and p=0.007 respectively) were found, and a negative correlation between age and native thiol/total thiol (p=0.007). In the hypoparathyroidism group, positive correlations between age and disulfide/native thiol and disulfide/total thiol ratios (p=0.017 and p=0.016, respectively) were found, and a negative correlation between age and native thiol/total thiol ratio (p=0.017) (Table 3).

**Table 3:** Factors related to oxidative stress parameters Age, BMI, and BUN, creatinine, eGFR and albumin levels were adjusted. (The analysis used partial correlation) BMI: Body mass index, cCa: Corrected calcium, p: Phosphor, Mg: Magnesium, PTH: Parathormone, 25 OH Vit. D: 25 hydroxy vitamin D, OSI: Oxidative stress index, TAS: Total Antioxidant Status, TOS: Total Oxidant Status, PON: Paraoxonase Enzyme activity, DS: Disulfide, TT: Total thiol, NT: Native thiol

Groups		Variables	TAS (mmol trolox equivalent/L)		TOS (lmol H_2_ O_2_ equivalent/L)		OSI (arbitrary unit)		PON (U/L)		NT (mmol/L)		TT (mmol/L)		DS (mmol/L)		DS/NT (%)		DS/TT (%)		NT/TT (%)	
			r	p	r	p	r	p	r	p	r	p	r	p	r	p	r	p	r	p	r	p
All participants		Age (years)	0.137	0.184	-0.013	0.898	-0.083	0.425	0.128	0.216	-0.306	0.003*	-0.265	0.010*	0.154	0.137	0.274	0.007*	0.275	0.007*	-0.274	0.007*
		(kg/m^2^)	0.317	0.008*	0.115	0.346	-0.011	0.926	0.159	0.192	-0.316	0.008	-0.281	0.019	-0.073	0.552	0.025	0.837	0.024	0.846	-0.025	0.836
		cCa (mmol/L)	-0.121	0.248	0.002	0.982	0.079	0.449	-0.123	0.241	-0.105	0.317	-0.144	0.168	-0.123	0.238	-0.129	0.219	-0.128	0.222	0.129	0.219
		P (mmol/L)	0.081	0.435	-0.092	0.374	-0.165	0.111	0.187	0.070	-0.073	0.483	0.004	0.968	0.265	0.009*	0.312	0.002*	0.311	0.002*	-0.310	0.002*
		Mg (mmol/L)	-0.013	0.904	0.034	0.746	0.041	0.696	0.022	0.836	0.005	0.960	0.000	1.000	0.083	0.425	0.031	0.766	0.032	0.761	-0.031	0.766
		PTH (ng/L)	0.045	0.665	0.189	0.066	0.259	0.011*	-0.126	0.224	-0.003	0.980	-0.068	0.511	-0.257	0.012*	-0.288	0.005*	-0.287	0.005*	0.288	0.005*
		25OH Vit. D (nmol/L)	-0.082	0.431	-0.122	0.237	-0.112	0.280	-0.097	0.350	0.054	0.604	0.011	0.919	-0.214	0.037*	-0.222	0.031*	-0.221	0.031*	0.221	0.032*
All patients		Age (years)	0.256	0.059	0.027	0.843	-0.104	0.449	0.249	0.067	-0.391	0.003*	-0.339	0.011*	0.118	0.391	0.321	0.017*	0.322	0.016*	-0.321	0.017*
		BMI (kg/m^2^)	0.357	0.012*	0.164	0.260	0.069	0.637	0.270	0.060	-0.279	0.052	-0.226	0.118	0.124	0.397	0.208	0.152	0.205	0.157	-0.207	0.154
		cCa (mmol/L)	-0.070	0.613	-0.028	0.842	-0.006	0.968	-0.243	0.074	-0.013	0.923	-0.004	0.980	0.091	0.507	0.087	0.530	0.087	0.528	-0.084	0.544
		P (mmol/L)	0.111	0.420	-0.036	0.794	-0.064	0.645	0.161	0.239	-0.221	0.105	-0.187	0.171	0.125	0.364	0.195	0.153	0.195	0.153	-0.193	0.159
		Mg (mmol/L)	0.087	0.529	0.265	0.050	0.270	0.046*	-0.137	0.317	-0.007	0.960	-0.003	0.981	0.109	0.427	0.061	0.658	0.065	0.639	-0.063	0.647
		PTH (ng/L)	0.096	0.487	0.101	0.464	0.090	0.514	-0.165	0.230	0.098	0.476	0.113	0.409	0.046	0.737	-0.007	0.958	-0.005	0.973	0.005	0.972
		25OH Vit.D (nmol/L)	-0.007	0.957	-0.0137	0.318	-0.182	0.183	-0.230	0.091	0.024	0.860	-0.016	0.907	-0.185	0.177	-0.179	0.190	-0.178	0.192	0.177	0.195
		cCa* (mmol/L)	0.053	0.737	0.052	0.739	0.045	0.772	-0.315	0.040*	0.147	0.348	0.188	0.226	0.277	0.072	0.202	0.195	0.201	0.196	-0.200	0.198*

There were positive correlations between disulfide, disulfide/native thiol and disulfide/total thiol ratios and the P and Mg values in all patients, and a negative correlation between PTH and 25 OH Vitamin D (Table 3).

For all participants, when the effects of age, BMI and BUN, creatinine, eGFR and albumin levels were adjusted in the partial correlation analysis, there were significant relationship between 25-OH vitamin D levels and DS, DS/NT, DS/TT and NT/TT (r=-0.287, p=0.024, r=-0.281, p=0.027, r=-0.293, p=0.021 and r=0.293, p=0.021, respectively).

In group hypoparathyroidism when the effects of age, BMI and BUN, creatinine, eGFR, and albumin levels were adjusted in the partial correlation analysis, there was a significant relationship between cCa levels and PON levels (r=-0.315, p=0.04).

In stepwise multivariate linear regression analysis, BMI and creatinine levels were found to be independent predictors of the TAS levels, cCa levels were found to be independent predictors of the PON levels and age was found to be an independent predictor of the NT and TT levels (Table 4).

**Table 4:** Stepwise multivariate linear regression analysis in the hypoparathyroidism group BMI: Body mass index, cCa: Corrected calcium, TAS: Total Antioxidant Status, PON: Paraoxonase Enzyme activity, TT: Total thiol, NT: Native thiol

	B	Std. Error	%95 CI	p	R^2^	p
TAS (mmolTrolox equivalent/L)					0.27	0.001
BMI (kg/m^2^)	0.012	0.004	0.003–0.02	0.01		
Creatinine (mmol/L)	0.672	0.217	0.236–1.109	0.003		
PON (U/L)					0.23	0.02
cCa (mmol/L)	-102.922	41.869	-187.303– -18.541	0.02		
NT (μmol/L)					0.16	0.004
Age (years)	-1.804	0.599	-3.01 – -0.59	0.004		
TT (μmol/L)					0.14	0.01
Age (years)	-1.779	0.659	-3.104 – -0.454	0.01		

## Discussion

To the best of our knowledge, this is the first study to demonstrate increased disulfide, disulfide/ native thiol, disulfide/total thiol in patients with hypoparathyroidism. In our study, it was found that the disulfide, disulfide/native thiol, disulfide/total thiol levels are higher in the hypoparathyroidism group than the control group. At the same time, a negative correlation between the 25 OH Vitamin D levels and these oxidant parameters, and another negative correlation between the serum calcium level and the PON level were found.

When the literature is reviewed, no studies that directly examined the relation between hypoparathyroidism and oxidative stress could be found. In our study, no statistically significant difference could be found between the two groups with regard to native thiol. The similarity of the TAS results in the patient and the control groups support that there has not been any change in the antioxidant system. In addition, the serum TOS levels were also found similar in both groups. However, when thiol/disulfide homeostasis was examined per se, it was observed that the disulfide and disulfide/native thiol ratio, which shifted towards the oxidant side of this scale, increased significantly. However, this increase in oxidation did not have an impact on the serum total oxidant level. We can relate the similarity of the TOS and TAS levels in the patient group to the accommodation of homeostasis with the activation of many antioxidant systems present in the metabolism [7] [16].

The increased disulfide levels in the patient group of our study could be related to the reactive oxygen radicals increasing due to hyperphosphatemia. Troyano et al. [17] showed that hyperphosphatemia had an effect via several mediators. In another study, increased reactive oxygen radicals were observed due to hyperphosphatemia induced decrease of nitric oxide release at the endothelium level [3]. Nguyen et al. [18] argued that there was mitochondrial oxidative stress in hyperphosphatemia. When the results of all these studies mentioned here and the results of our study are examined, we can argue that the shift towards the disulfide in the hypoparathyroidism could be related to age, hyperphosphatemia, and changes that we could not determine at the endothelium level. Further in vitro and in vivo studies are required to make explicit comments on this topic.

It has been shown in various studies in the literature that vitamin D deficiency causes a predisposition to oxidative stress [19] [20]. In studies with children, it was seen that vitamin D supplement caused a significant amelioration in the antioxidant system [21]. Martins et al. [22] pointed out to there was an increase in the PTH levels with vitamin D supplement, and also in several inflammatory biomarkers such as TNF alpha and IL 8. Similarly, a decrease in the PTH level was observed after administering active vitamin D (calcitriol) to hemodialysis patients, accompanied by a decrease in inflammatory markers such as C reactive protein (CRP) and interleukin 6 (IL 6) and an increase in anti-inflammatory markers (CD 4 + IL 4) [23]. In our study too, a negative correlation was observed between vitamin D level and the disulfide/thiol and disulfide levels supporting the literature. The positive effect of vitamin D and calcium supplements on antioxidant capacity in women with vitamin D deficiency was shown [24] [25]. Considering the results of both these studies and our study, it was seen that vitamin D plays an active role in the antioxidant system. Although no correlation between the TAS level and the 25-OH vitamin D level could be found in our data, we think that monitoring 25-OH vitamin D levels and replacement of vitamin D in patients with hypoparathyroidism follow-ups is important due to the correlation between the disulfide and the disulfide/thiol ratio.

In recent studies, although elevated PTH is correlated with oxidative stress [26] [27], low PTH levels were found related to oxidative stress [28] [29]. It was shown, in a cell culture study, that parathyroid hormone-related peptide (PTHrP) prevented the proapoptotic effects of reactive oxygen radicals in osteoblasts over MKP1 dephosphorylation [30]. In a study conducted on mice, it was found that downregulation of MKP1 provided strong protection against oxidative stress, and PTHrP was cardioprotective most probably via downregulation of MKP-1 and the activation of the MAPK and PI3K/AKT signals. In that study, it was found that a PTHrP application before the mice had been exposed to oxidative damage via H_2_O_2_ , reduced cardiomyocyte damage [31]. In our study, the negative correlation between the PTH level and the disulfide and disulfide/thiol ratios showed that hypoparathyroidism is a state of oxidative stress. However, in another study examining the relation of PTH to inflammation and oxidative stress, no correlation could be found. The patient population in that study comprised patients with chronic kidney deficiency receiving hemodialysis treatment, and it was argued that the oxidative stress markers (total plasma antioxidant capacity, malonic dialdehyde, lipid hydroperoxidation, advanced oxidation protein products, quantification of nitric oxide metabolites, 8-isoprostane) and inflammation markers (interleukins 1 and 6, tumour necrosis factor-alpha) were related to other mechanisms independent from PTH [32]. In light of all this information, we assume that the normal level of PTH is one of the elements maintaining the oxidant balance.

In our study, a negative correlation was found between PON and cCa. PON1 is known to have effects on preventing cardiovascular diseases and atherosclerosis [12]. In other words, it is seen that low Ca levels shifted the oxidant balance towards the antioxidant side when compared to high Ca level. However, in Yarmohammadi et al. [2], blood Ca levels lower than 2.23 mmol/L were found to relate to 2.3 times higher sudden cardiac deaths when compared with blood Ca levels higher than 2.38 mmol/L. It becomes difficult to make inferences from such results since atherosclerosis and sudden cardiac deaths are affected by multi-parameters variables. For instance, PTH increases aldosterone release through indirect paths. The increased cardiovascular disease risk due to lowered aldosterone levels after a parathyroidectomy could be explained through this mechanism [33]. The low calcium level in our findings might have increased the PON levels through the antioxidant path to normalize the oxidative balance, which had been shifted to the oxidant side in hypoparathyroidism patients.

In our study, when both the patient group and all patients were examined as separate groups, the increase in disulfide/native thiol and disulfide/total thiol ratios with aging attracted attention. It is assumed that aging is caused by free oxygen radicals gradually increasing in the body. However, as the favourable effects of free oxygen radicals on especially several immune events have been examined recently, it is understood that they also have pro-survival effects [34]. We hope that the exciting lack of information would be filled in very soon.

Another result we found in our study was the positive correlation between the BMI and native and total thiol levels. In fact, it is expected that the disulfide level increase with the thiol oxidation as the degree of obesity increase [15]. The negative correlation between the BMI and the native and total thiol levels in our results supports this expectation. However, quite surprisingly, the positive correlation between the BMI and TAS suggests that although the oxidative paths related to increasing bodyweight of the organism are activated, several antioxidant materials can also increase with the compensatory mechanisms. In a study conducted with obese children between 7 and 14 years of age, it was found that they have lower vitamin D levels and higher PTH levels compared to the healthy control group, and the oxidative stress markers (malondialdehyde, myeloperoxidase, and 3-nitrotyrosine) and the inflammatory markers were higher [35]. That study suggests that obesity lowered vitamin D levels and elevated PTH together disturbed the balance in favour of the oxidant system. In our study, the absence of elevated PTH and even similar results from patients with low PTH increased the reliability of the result obtained.

In a very recent study, dynamic thiol/disulfide balance was shown to be affected by smoking [36]. In our study, there were no smoking patients. Thus, we eliminated this misleading factor and increased the reliability of the disulfide-thiol balance results.

In previous studies, it was shown that thyroid function disorder could increase the oxidative stress parameters [13] [16]. However, Kaçmaz et al. [29] found that oxidative stress mediators (malondialdehyde, nitric oxide) increased following thyroparathyroidectomy despite the replacement treatment. Therefore, the data about this topic is controversial. The thyroid functions of the patients were not examined in our study. Thus, we see this as a limitation in the interpretation of the data.

Due to the total number of participants in our study, the generalizability of our results seems weak. Randomized controlled studies with a higher number of participants are required.

We think that our study may provide a path to other studies that can be conducted on this topic with regard to examining the hypoparathyroidism and oxidative stress relation using different markers.

## Conclusion

Parathormone has a crucial role in maintaining and sustaining the oxidant balance. Hypoparathyroidism causes the oxidation of the thiol groups of the proteins, and induces the formation of disulfide bonds and creates a predisposition to oxidative stress. The decrease in the 25-OH vitamin D level and the increase in BMI also predict oxidative stress. For these reasons, the cCa and 25-OH vitamin D levels should be paid attention to and kept in the normal range in patients with iatrogenic hypoparathyroidism, a frequent reason for parathyroidism.

### Conflict of interest statement

The authors stated that they have no conflicts of interest regarding the publication of this article.

## References

[1] Lopes M P, Kliemann B S, Bini I B, Kulchetscki R, Borsani V, Savi L, Borba V Z C, Moreira C A (2016). Hypoparathyroidism and pseudohypoparathyroidism: Etiology, laboratory features and complications. Archives of Endocrinology and Metabolism.

[2] Yarmohammadi H, Uy-Evanado A, Reinier K, Rusinaru C, Chugh H, Jui J, Chugh S S (2017). Serum Calcium and Risk of Sudden Cardiac Arrest in the General Population. Mayo Clin Proc.

[3] Iijima K (2012). Hyperphosphatemia and cardiovascular diseases: Impact of vascular calcification and endothelial dysfunction. Clin Calcium.

[4] Liu H, Zhang J (2012). Cerebral Hypoperfusion and Cognitive Impairment: The Pathogenic Role of Vascular Oxidative Stress. Int J Neurosci.

[5] Krata N, Zagożdżon R, Foroncewicz B, Mucha K (2018). Oxidative Stress in Kidney Diseases: The Cause or the Consequence?. Arch Immunol Ther Exp (Warsz).

[6] Kopáni M, Celec P, Danišovič L, Michalka P, Biró C (2006). Oxidative stress and electron spin resonance. Clin Chim Acta.

[7] Aslan R, Kutlu R, Civi S, Tasyurek E (2014). The correlation of the total antioxidant status (TAS), total oxidant status (TOS) and paraoxonase activity (PON1) with smoking. Clin Biochem.

[8] Pitocco D, Tesauro M, Alessandro R, Ghirlanda G R, Cardillo C (2013). Oxidative Stress in Diabetes: Implications for Vascular and Other Complications. Int J Mol Sci.

[9] Erel O (2004). A novel automated direct measurement method for total antioxidant capacity using a new generation, more stable ABTS radical cation. Clin Biochem.

[10] Klisić A, Kocić G, Kavarić N, Pavlović R, Soldatović I, Ninić A (2019). Nitric oxide products are not associated with metabolic syndrome. J Med Biochem.

[11] van Himbergen T M, van Tits L J, Roest M, Stalenhoef A F (2006). The story of PON1: How an organophosphatehydrolysing enzyme is becoming a player in cardiovascular medicine. Neth J Med.

[12] Costa L G, Vitalone A, Cole T B, Furlong C E (2005). Modulation of paraoxonase (PON1) activity. Biochem Pharmacol.

[13] Ates I, Altay M, Yilmaz F M, Topcuoglu C, Neselioglu S, Erel O, Yilmaz N M (2016). Dynamic thiol/disulfide homeostasis in patients with autoimmune subclinical hypothyroidism. Endocr Res.

[14] Ulas T, Buyukhatipoglu H, Kirhan I, Dal M S, Ulas S, Demir M E, Eren M A, Ucar M, Hazar A, Kurkcuoglu İ C, Aksoy N (2013). Evaluation of oxidative stress parameters and metabolic activities of nurses working day and night shifts. Revista da Escola de Enfermagem da USP.

[15] Erel O, Neselioglu S (2014). A novel and automated assay for thiol/disulphide homeostasis. Clin Biochem.

[16] Ates I, Arikan M F, Altay M, Yilmaz F M, Yilmaz N M, Berker D, Guler S (2018). The effect of oxidative stress on the progression of Hashimoto's thyroiditis. Arch Physiol Biochem.

[17] Troyano N, Nogal M D, Mora I, Diaz-Naves M, Lopez-Carrillo N, Sosa P, Rodriguez-Puyol D, Olmos G, Ruiz-Torres M P (2015). Hyperphosphatemia induces cellular senescence in human aorta smooth muscle cells through integrin linked kinase (ILK) up-regulation. Mech Ageing Dev.

[18] Nguyen T T, Quan X, Hwang K H, Xu S, Das R, Choi S K, Wiederkehr A, Wollheim C B, Cha S, Park K (2015). Mitochondrial oxidative stress mediates high-phosphate-induced secretory defects and apoptosis in insulin-secreting cells. Am J Physiol Endocrinol Metab.

[19] Tao S, Yuan Q, Mao L, Chen F L, Ji F, Cui Z F (2017). Vitamin D deficiency causes insulin resistance by provoking oxidative stress in hepatocytes. Oncotarget.

[20] Damiati S (2019). A pilot study to assess Kidney functions and toxic dimethyl-arginines as risk biomarkers in women with low vitamin D levels. J Med Biochem.

[21] Doğan M, Cesur Y, Zehra D Ş, Kaba S, Bulan K, Cemek M (2012). Oxidant/antioxidant system markers and trace element levels in children with nutritional rickets. J Pediatr Endocrinol Metab.

[22] Martins D, Meng Y X, Tareen N, Artaza J, Lee J E, Farodolu C, Gibbons G, Norris K (2014). The Effect of Short Term Vitamin D Supplementation on the Inflammatory and Oxidative Mediators of Arterial Stiffness. Health (Irvine Calif).

[23] Wu C C, Chang J H, Chen C C, Su S B, Yang L K, Ma W Y, Zheng C M, Diang L, Lu K (2011). Calcitriol Treatment Attenuates Inflammation and Oxidative Stress in Hemodialysis Patients with Secondary Hyperparathyroidism. Tohoku J Exp Med.

[24] Razavi M, Jamilian M, Karamali M, Bahmani F, Aghadavod E, Asemi Z (2016). The Effects of Vitamin D-K-Calcium Co-Supplementation on Endocrine, Inflammation, and Oxidative Stress Biomarkers in Vitamin D-Deficient Women with Polycystic Ovary Syndrome: A Randomized, Double-Blind, Placebo-Controlled Trial. Horm Metab Res.

[25] Foroozanfard F, Jamilian M, Bahmani F, Talaee R, Talaee N, Hashemi T, Nasri K, Asemi Z, Esmaillzadeh A (2015). Calcium plus vitamin D supplementation influences biomarkers of inflammation and oxidative stress in overweight and vitamin D-deficient women with polycystic ovary syndrome: A randomized double-blind placebo-controlled clinical trial. Clin Endocrinol (Oxf).

[26] Vidal A, Sun Y, Bhattacharya S K, Ahokas R A, Gerling I C, Weber K T (2006). Calcium paradox of aldosteronism and the role of the parathyroid glands. Am J Physiol Heart Circ Physiol.

[27] Tanaka M, Tokunaga K, Maruyama T, Otagiri M, Tominaga Y, Itoh K, Matsushita K, Komaba H, Fukagawa M (2011). Parathyroidectomy Markedly Reduces Oxidative Stress in a Patient with Primary Hyperparathyroidism. Ther Apher Dial.

[28] Pecoits-Filho R, Lindholm B, Stenvinkel P (2002). The malnutrition, inflammation, and atherosclerosis (MIA) syndrome: The heart of the matter. Nephrol Dial Transplant.

[29] Kaçmaz M, Atmaca M, Arslan A, Demir H, Özbay M F (2015). Oxidative stress in patients with thyroidectomy and thyroparathyroidectomy under replacement therapy. Endocrine.

[30] Ardura J A, Portal-Núñez S, Castelbón-Calvo I, Martínez D T I, de la Fuente M, Esbrit P (2017). Parathyroid Hormone-Related Protein Protects Osteoblastic Cells from Oxidative Stress by Activation of MKP1 Phosphatase. J Cell Physiol.

[31] Datta N S, Chukkapalli S, Vengalil N, Zhan E, Przyklenk K, Lasley R (2015). Parathyroid hormone-related peptide protects cardiomyocytes from oxidative stress-induced cell death: First evidence of a novel endocrine-cardiovascular interaction. Biochem Biophys Res Commun.

[32] Jaqueto M, Delfino V D A, Bortolasci C C, Barbosa D S, Morimoto H K, Frange R F N, Ferreira L F F, Guimarães F B D S (2016). Are PTH levels related to oxidative stress and inflammation in chronic kidney disease patients on hemodialysis?. J Bras Nefrol.

[33] Tomaschitz A, Ritz E, Pieske B, Rus-Machan J, Kienreich K, Verheyen N, Gaksch M, Grübler M, Fahrleitner-Pammer A, Mrak P, Toplak H, Kraigher-Krainer E, März W, Pilz S (2014). Aldosterone and parathyroid hormone interactions as mediators of metabolic and cardiovascular disease. Metabolism.

[34] Liochev S I (2013). Reactive oxygen species and the free radical theory of aging. Free Radic Biol Med.

[35] Codoñer-Franch P, Tavárez-Alonso S, Simó-Jordá R, Laporta-Martín P, Carratalá-Calvo A, Alonso-Iglesias E (2012). Vitamin D Status is Linked to Biomarkers of Oxidative Stress, Inflammation, and Endothelial Activation in Obese Children. J Pediatr.

[36] Solak I, Cetinkaya C D, Gederet Y T, Kozanhan B, Erel O, Eryilmaz M A (2018). Effects of smoking on thiol/disulfide homeostasis. Eur Rev Med Pharmacol Sci.

